# Supplementary material for: Mind In Vitro Platforms: Versatile, Scalable, Robust, and Open Solutions to Interfacing with Living Neurons
Source: Adv Sci (Weinh). 2023 Dec 31;11(11):2306826. doi: 10.1002/advs.202306826 (PMC10953569; doi:10.1002/advs.202306826)
Supplement: Supplementary file 1 — Supporting Information [file ADVS-11-2306826-s001.pdf]

ADVANCED SCIENCE
Open Access

Supporting Information

for *Adv. Sci.*, DOI 10.1002/advs.202306826

Mind In Vitro Platforms: Versatile, Scalable, Robust, and Open Solutions to Interfacing with Living Neurons

*Xiaotian Zhang, Zhi Dou, Seung Hyun Kim, Gaurav Upadhyay, Daniel Havert, Sehong Kang, Kimia Kazemi, Kai-Yu Huang, Onur Aydin, Raymond Huang, Saeedur Rahman, Austin Ellis-Mohr, Hayden A. Noblet, Ki H. Lim, Hee Jung Chung, Howard J. Gritton, M. Taher A. Saif, Hyun Joon Kong, John M. Beggs and Mattia Gazzola**

Supplementary Information for 'Mind *in Vitro*' platforms: Versatile, scalable, robust and open solutions to interfacing with living neurons

Xiaotian Zhang, Zhi Dou, Seung-Hyun Kim, Gaurav Upadhyay, Daniel Havert, Sehong Kang, Kimia Kazemi, Kai-Yu Huang, Onur Aydin, Raymond Huang, Saeedur Rahman, Austin Ellis-Mohr, Hayden A. Noblet, Ki H. Lim, Hee Jung Chung, Howard J. Gritton, M. Taher A. Saif, Hyun Joon Kong, John M. Beggs, Mattia Gazzola*

Xiaotian Zhang, Onur Aydin, Hee Jung Chung, M. Taher A. Saif, Hyun Joon Kong and Mattia Gazzola
Carl R. Woese Institute for Genomic Biology, University of Illinois at Urbana–Champaign
Urbana, IL 61801
Email Address: mgazzola@illinois.edu

Zhi Dou, Seung-Hyun Kim, Gaurav Upadhyay, Sehong Kang, Kimia Kazemi,
Raymond Huang, Saeedur Rahman, M. Taher A. Saif and Mattia Gazzola
Department of Mechanical Science and Engineering, University of Illinois at Urbana–Champaign
Urbana, IL 61801

Daniel Havert and John M. Beggs
Department of Physics, Indiana University Bloomington
Bloomington, IN 47405

Kai-Yu Huang and Hyun Joon Kong
Department of Chemical and Biomolecular Engineering, University of Illinois at Urbana–Champaign
Urbana, IL 61801

Austin Ellis-Mohr
Department of Electrical and Computer Engineering, University of Illinois at Urbana–Champaign
Urbana, IL 61801

Hayden A. Noblet, Ki H. Lim and Hee Jung Chung
Molecular and Integrative Physiology, University of Illinois at Urbana–Champaign
Urbana, IL 61801

Hayden A. Noblet and Hee Jung Chung
Neuroscience Program, University of Illinois at Urbana–Champaign
Urbana, IL 61801

Hee Jung Chung and Howard J. Gritton
Beckman Institute for Advanced Science and Technology, University of Illinois at Urbana–Champaign
Urbana, IL 61801

Howard J. Gritton
Department of Comparative Biosciences, University of Illinois at Urbana–Champaign
Urbana, IL 61802

Supplementary Note 1 - Hardware Components

In this section, we discuss in detail our hardware construction. In Table S1, we list all the hardware components required to construct a system with recording capacity of up to 512 channels, assuming that Open Ephys board is utilized for downstream data acquisition. The cost of each component is listed in Table S1. We note that the Open Ephys board and Intan headstages have higher cost relative to the rest of the components on the list. However, both the Open Ephys board and the Intan headstages are open-source and can be obtained at a lower cost by self-assembling the individual electronic constituents [1, 2], providing the opportunity to reduce the overall cost of each device by ∼50% (costs in parentheses).

Table S1: List of components used in constructing the recording hardware along with their costs in USD.[a]

Component	Cost (USD)	Component	Cost (USD)
Open Ephys board<br>(self-assemble[b])	3200<br>(∼1000)	Intan RHD 32ch Headstage<br>(self-assemble)	940<br>(∼600)
Intan RHD 64ch Headstage<br>(self-assemble)	1875<br>(∼800)	Intan RHD 128ch Headstage<br>(self-assemble)	2995<br>(∼1200)
Omnetics connector[c]	85.21	Molex connector[d]	1
36-pin adapter board	255	RHD SPI cable	215
RHD dual adaptor	345	POGO pins[e]	∼0.6
Other hardware[f]	∼60		

[a] Online prices as of Aug 2023. [b] Self-assemble cost estimates only account for raw material costs, excluding external chip assembling service. [c] Omnectics 36 position dual row nano strip A79022-001. [d] Molex SlimStack 5024306410. [e] Mill-Max spring-loaded pogo pins with regular tail length 0914-2-15-20-77-14-11-0, with extended tail for electrical stimulation 0929-0-15-20-75-14-11-0. [f] Other hardware includes acrylic boards, hinge, latch, bumpers and PCB fabrication. We fabricate our PCBs externally through Oshpark LLC.

As underscored in the main text, our hardware platform is modularly designed to support a range of recording configurations with 59, 128, 256 and 512 channels. While Table S1 showcases the aggregation of all components utilized in these configurations, only a subset of the list is necessary to assemble a specific prototype. As shown in Table S1, a series of Intan (RHD series) headstages are available with various recording capacities (32, 64, 128). In principle, users are free to select any combination of these headstages for each recording configuration. However, we note that different headstages may require different connectors and PCB footprints for interfacing with the MEA. In this study, we have employed three different connector series: Intan adapter board, Omnetics Nano Strip connector and Molex SlimStack connector, listed in descending order of cost. A trade-off is then found between cost and assembly difficulty: the more inexpensive options generally require finer surface mount soldering technique, which in turn results in additional manufacturing time. Therefore, to strike a balance between cost and manufacturing complexity, we present two different headstage and connector selections for each configuration in Table S2. Both options are natively compatible with our recording platform and similar recording qualities are observed during testing. Therefore, we provide PCB designs involving footprints for all three connector series, so that users can select based on their fabrication capability, and adopt these designs for their specific needs [3].

Table S2: Headstage, connector and cable selections to construct 59, 128, 256 and 512-channel recording systems. Total cost of each configuration includes the costs of the components on the list (headstage, connector and cable), an Open Ephys data acquisition board, the required amount of pogo pins and other hardware accessories needed to construct the recording platform.

Config.	Headstage options	Connector options	Cable options	Total cost (USD)	*Total cost self-assembly (USD)*
59ch	2×RHD 32	2×adapter board	2×SPI	∼6k	*∼3.5k*
	1×RHD 64	2×Omnetics	1×SPI	∼5.5k	*∼2.3k*
128ch	2×RHD 64	4×Omnetics	2×SPI	∼7.7k	*∼3.6k*
	1×RHD 128	2×Molex	1×SPI	∼6.5k	*∼2.6k*
256ch	4×RHD 64	8×Omnetics	4×SPI	∼12k	*∼6k*
	2×RHD 128	4×Molex	2×SPI	∼9.7k	*∼4.1k*
512ch	8×RHD 64	16×Omnetics	4×(SPI+dual adapter)	∼22k	*∼11.5k*
	4×RHD 128	8×Molex	4×SPI	∼16k	*∼7k*

Finally, we include the list of components required for employing Intan bi-directional recording/stimulation controller in Table S3. As discussed in the main text, this controller is an alternative for downstream data collection if high resolution electrical stimulation is desired. We note a different series of headstage and SPI cable (RHS series) are needed here for bi-directional signal communication. Nevertheless, the RHS 32 headstage em-

ploys the same connector scheme as the RHD 32 counterpart, so that they are fully interchangeable in terms of interfacing with the PCB.

Table S3: List of components used for bi-directional recording/stimulation system along with their costs in USD.[a]

Component	Cost	Component	Cost
RHS stimulation/recording controller	11800	Intan RHS 32ch Headstage	1485
RHS SPI cable	265		

[a] Online prices as of Aug 2023

Supplementary Note 2 - Comparison with HD-MEA

In Figure S1, we present a detailed comparison between our system and CMOS-based, commercial high density-MEA (HD-MEA) solutions.

Specifications	Maxwell MaxOne	3Brain BioCAM DupleX	Multichannel systems MEA5000	**This Work**
System type	Active - CMOS	Active - CMOS	Active - CMOS	Passive
Electrode number	26400	4096	4225 (Rec) + 1024 (Stim)	up to 512
Recording channels	1020	4096	4225	512
Stimulation channels	32	4	3	128
MEA configuration	Fixed	Fixed	Fixed	Customizable
System reconfigurability (Hardware desgin; single to multi-well)	No	No	No	Yes
Open-source software (Full functionality freely accessible)	No (No, additional license fee)	No (No, additional license fee)	No (Yes)	Yes
Open-source systems	No	No	No	Yes
Cost to start experiment (system + software + MEA)	~$100k	~$100k	~$100k	~$10k

Figure S1: Comparison between our customized, passive electrophysiology solution with commercial HD-MEA (high-density MEA) systems. Costs are based on acquired quotations.

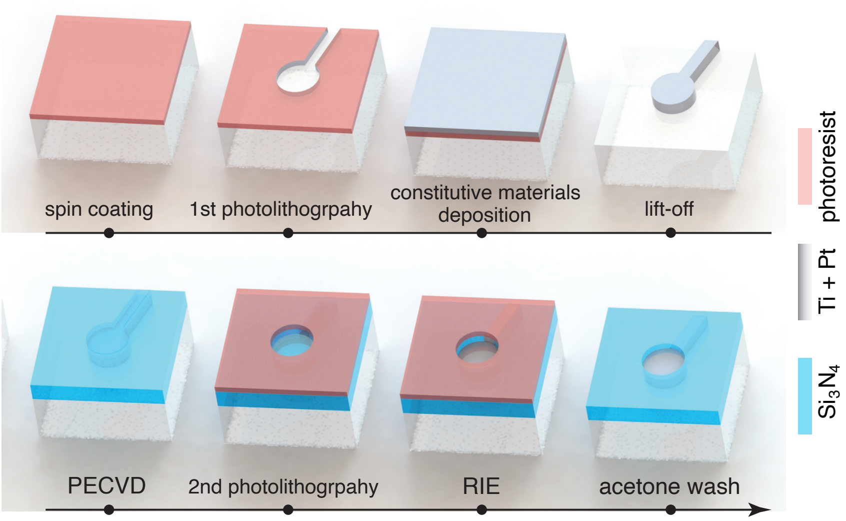

Figure S2: Illustration of the MEA micro-fabrication process using standard cleanroom technologies.

Supplementary Note 3 - MEA Fabrication

In Figure S2, we illustrate our MEA fabrication process. Key information related to the machine and parameter settings are described in the Experimental Section of the main text. A more comprehensive instruction and protocol of our process is publicly available [3].

In Section 3 of the main text, we characterize the impedance of our custom MEAs across a variety of electrode diameters. This characterization is performed on a specially designed 59-electrode MEA, where electrodes of four different sizes are arranged in separate quadrants. Microscopic image of this MEA is presented in Figure S3, demonstrating electrode with diameters of 5, 10, 20 and 30μm, respectively. The effect of Pt deposition on each electrode diameter is also demonstrated in the figure.

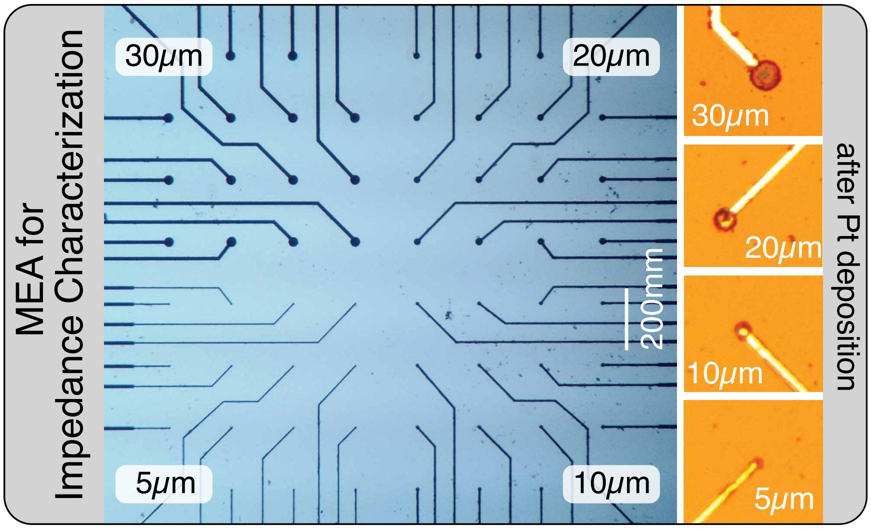

Figure S3: Image of the MEA with four different electrode sizes used for impedance characterization.

Supplementary Note 4 - MEA Employment

In this section, we present more details about employing our MEAs for recording. In Figure S4a, we illustrate the PDMS culture wells affixed on the MEA for seeding 2D neural cultures or 3D engineered tissues. The size and shape of these wells can be customized depending on the electrode arrangement, but they are generally larger than the area spanned by the electrode pattern. Finally, a glass ring is also glued on the MEA for holding culture media. In Figure S4b, we demonstrate the use of 3D-printed stoppers for centering and holding MEAs of various sizes and shapes. This ensures a precise and reproducible loading and alignment of the chips, enhancing recording quality.

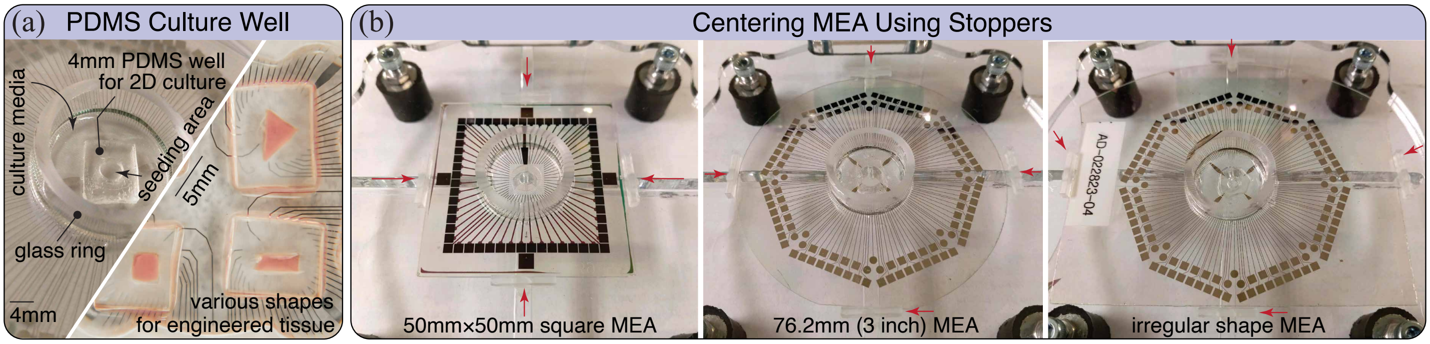

Figure S4: (a) PDMS wells of various shapes for neuron seeding. (b) Using 3D-printed stoppers for centering different MEAs.

Supplementary Note 5 - System Characterization with Primary Neurons

To demonstrate the recording capability and characterize the performance of our system, we present result and analysis from embryonic stem cell-derived motor neurons (ESC-MNs) in Section 4.1 of main text. To further showcase a consistent recording quality across different cell types, in this section, we present the same characterizations with primary neuron (PN) cultures.

With the utility of the rectangle and curved MEA layouts discussed in Section 4 of main text, we now employ the perturbed layout of the 128-channel MEA (Figure 3c of main text) for culturing PN cells in this experiment.

The spontaneous activity of the cultured sample (Figure S5a) is visualized through the raster plot of Figure S5b. Snapshots of the filtered signals are also plotted for representative channels, illustrating the detectability of both single unit activities as well as synchronized bursts events. We then quantify the recording quality by characterizing signal-to-noise ratio (SNR) across channels (Figure S5c). Similar to the results from ESC-MNs (Figure 4d), a SNR>5 is observed for almost all channels. Finally, we demonstrate the electrical stimulation of the culture by employing the same protocol described in Figure 4e, where biphasic electric pulses are applied at either side of the culture for selective activation. The heat-maps in Figure S5d showcase the average firing rate of each electrode, while providing visualizations of the spatial activity pattern in response to stimulation at different sites. Overall, through this experiment, consistent recording and stimulation characteristics are observed with PN cultures as in the case of ESC-MNs, demonstrating the stability and consistency of our system performance across different cell types.

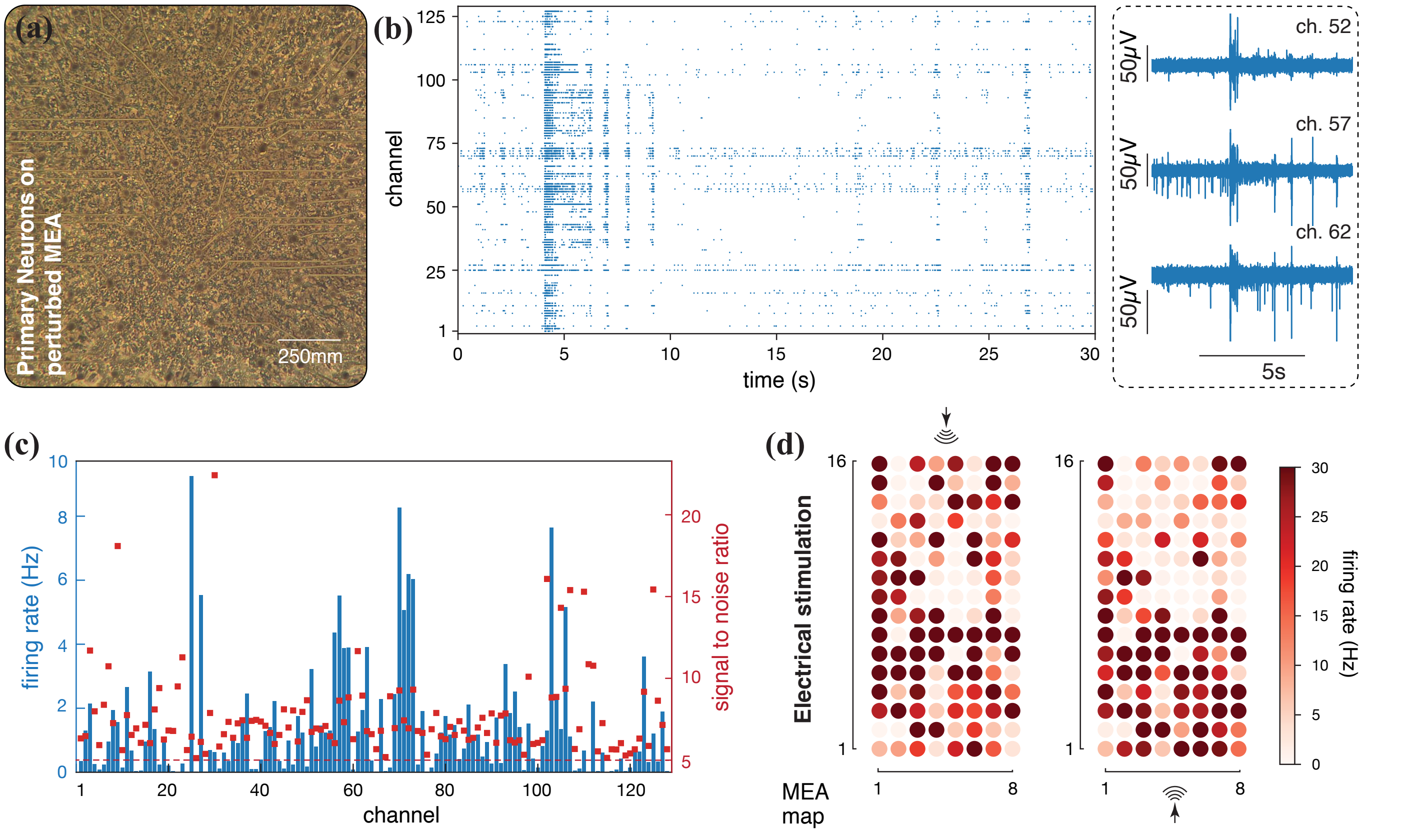

Figure S5: (a) Microscopic image of PNs seeded on a 128-channel MEA with perturbed layout and 30μm electrodes. **(b)** Raster plot shows the spontaneous neural activity recorded 15 days after seeding. Snapshots of filtered data from representative channels. **(c)** Averaged firing rate and SNR of each recording channel on day 15. **(d)** Spatial activity propagation in response to localized electrical stimulation at two different stimulation sites (top and bottom). Stimulation is also performed on day 15, with intensity of 1500mV.

Supplementary Note 6 - ITO MEA Characterization

The reconfigurability of our hardware system provides the opportunity of integrating electrophysiology with advanced microscopy. This is demonstrated in Section 4.3 of the main text, where primary neurons are seeded on transparent, indium tin oxide (ITO) MEAs (Figure S6a) for concurrent calcium imaging and electrophysiology recording. We fabricate the ITO MEA following the process presented in Figure S2, and then characterize its transmittance using an ultraviolet–visible spectrophotometer (UV-Vis, Varian Cary5G). To this end, an aperture plate is mounted onto the ITO MEA, and then aligned so that one of the contact pads is fully covering the aperture. Results presented in Figure S6b showcase that our ITO MEA consistently exhibits a transmittance of >80% for a range of visible spectrum wavelengths between 400 to 800nm, making it suitable for fluorescence imaging of neurons [4].

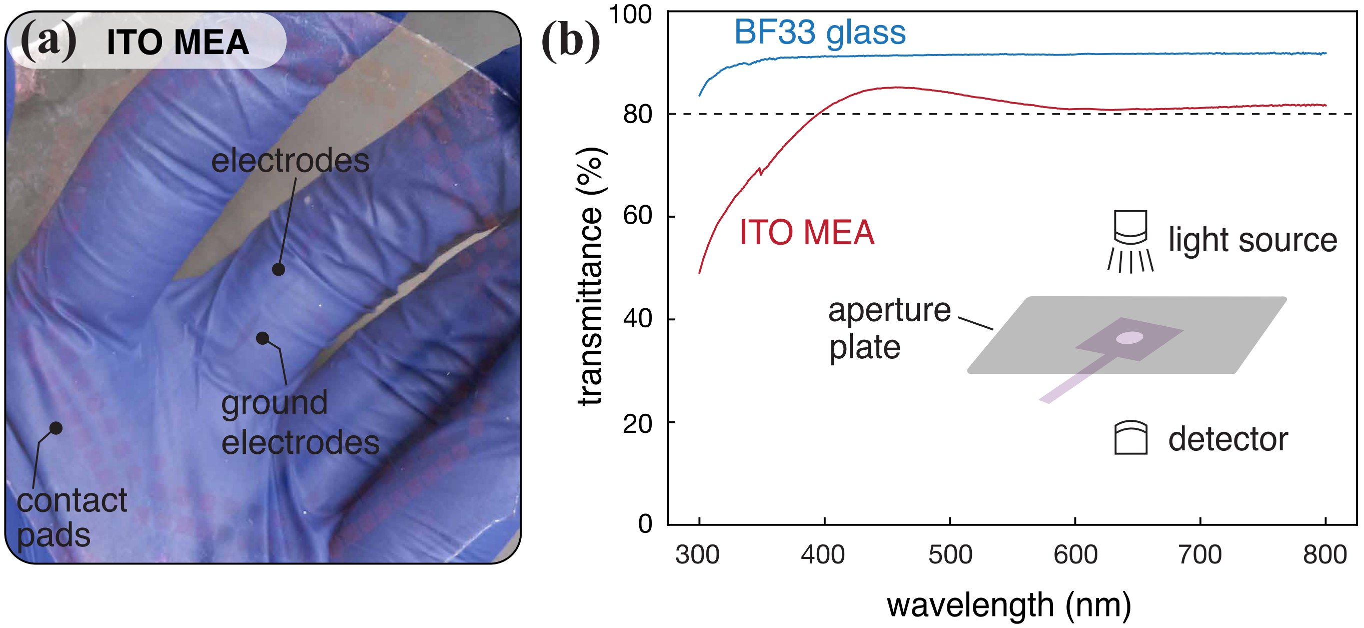

Figure S6: (a) Transparent MEA with 100nm ITO layer deposited on a BOROFLOAT 33 (BF33) glass wafer. (b) Transmittance of the ITO MEA at different wavelengths. Measurements of the plain glass wafer are also plotted for comparison.

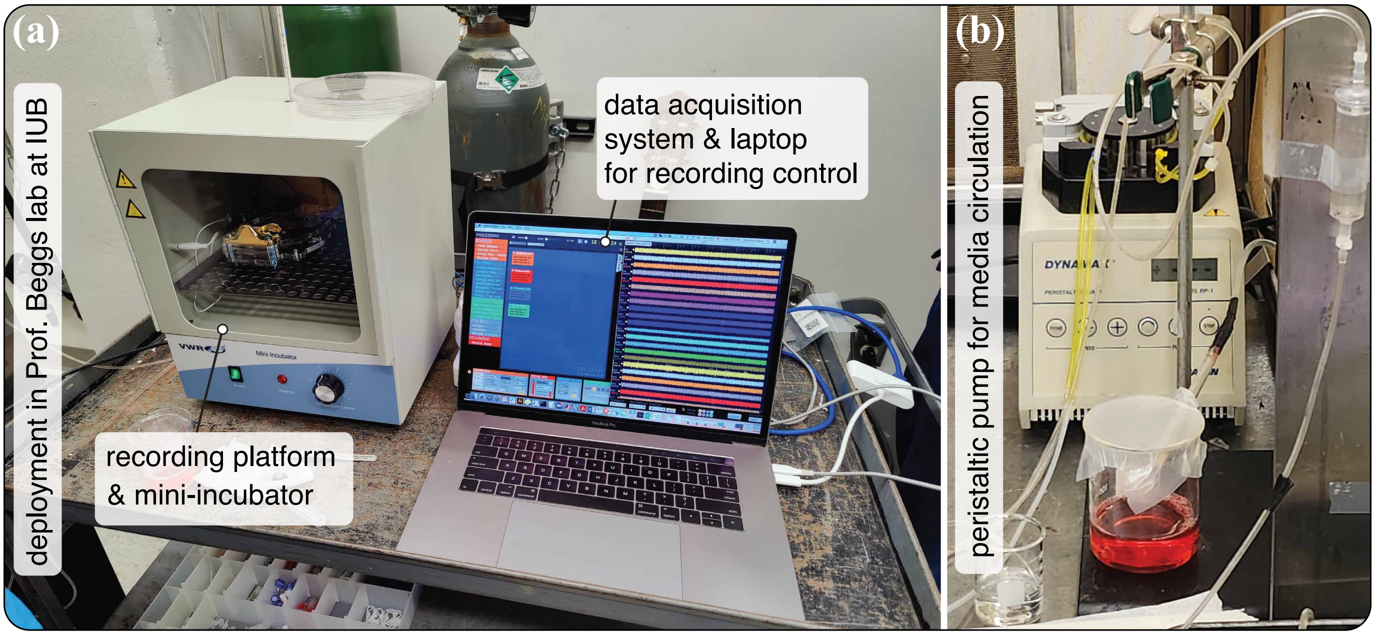

Figure S7: (a) Deployment of our system in Prof. Beggs lab at Indiana University Bloomington. (b) Connecting the recording platform with a dual channel peristaltic pump for culture media circulation.

Supplementary Note 7 - Portability and Reproducibility

While our system was mainly developed at University of Illinois Urbana-Champaign, we demonstrate its portability through off-site recordings of organotypic brain slices that are cultured and prepared at Indiana University Bloomington. To this end, essential equipment is transported, including the Open Ephys board, a 128-channel hardware platform, multiple MEAs, a mini-incubator and a laptop. These devices demand minimal lab-space and can be conveniently set up with a very small footprint (Figure S7a). For long-term or organotypic tissue recordings where maintaining oxygen and pH balance are critical, our system is fully compatible with systems optimized for media exchange. This is achieved through connecting the fluidic interface of Figure 5a in the main text to the peristaltic pump depicted of Figure S7b. We note that this fluidic system is primarily designed for organotypic tissue experiments, in which samples are not preserved after one day of recording. In case of experiments involving cell cultures and much longer monitoring (weeks-long), the media refreshment system has to be

carefully redesigned to ensure robustness, reliability, and a sterilized environment throughout the entire process. Although it is out of the scope of this paper, we are actively working on delivering such system as an extension of our current setup, enabling continuous, multi-day experiments across diverse culture types.

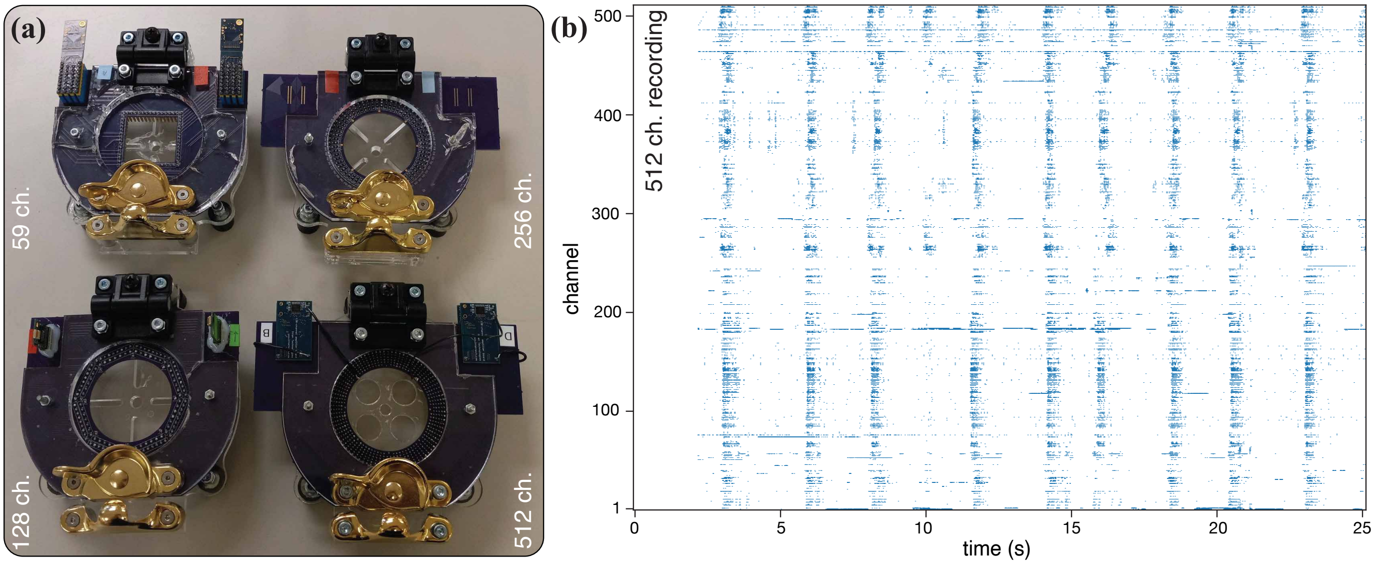

Figure S8: (a) Four recording platforms with increasing number of recording channels. (b) Spontaneous recordings using the 512-channel device and MEA.

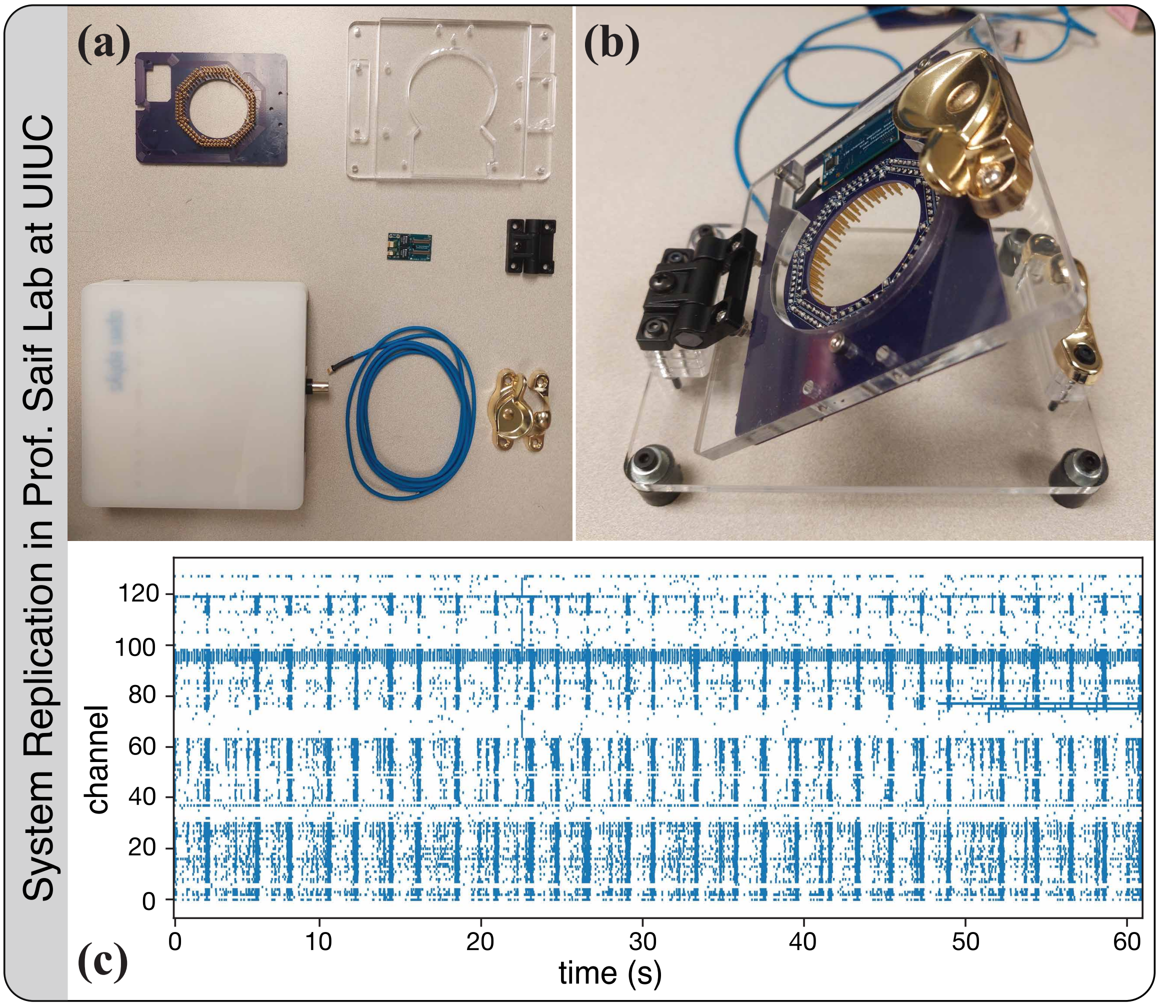

Figure S9: (a),(b) System replication and deployment at Prof. Saif lab at UIUC. (c) System validation through the recording of the spontaneous activity of an ESC-MNs culture.

Apart from portability, we further demonstrate that our system is easily reproducible. Leveraging our shared designs and protocols, a set of four different platforms built by different investigators is shown in Figure S8. Each one hosts a different PCB interface, demonstrated in recording systems with 59, 128, 256 and 512 channels, respectively. Moreover, with the capability of the 128 channel version extensively discussed in the main text, here, we showcase the maximum recording capacity of our system. To this end, the 512-electrode MEA presented in Figure 3b is seeded with ESC-MNs for testing. A 512 channel raster plot is presented in Figure S8b, illustrating the spontaneous activity of the culture on day 7 after seeding. As can be seen from the plot, spikes and synchronized bursts are detected for almost all recording channels, demonstrating the stability and the functionality of our system at the maximum recording capacity.

Finally, we present an example of our system being reproduced by researchers with no previous experience. Figure S9a,b showcase a replica of our 128-channel recording solution developed in Prof. Saif lab at UIUC. Following our open-protocol, this system is assembled with a total amount of 15 hours of work, and successfully validated with a ESC-MNs culture. Recordings carried out by this new system are presented in Figure S9c.

References

[1] https://open-ephys.github.io/acq-board-docs/ .

[2] https://intantech.com .

[3] Open-source Hardware: https://gazzolalab.github.io/MiV-OH/; Software: https://miv-os.readthedocs.io .

[4] S. Middya, V. F. Curto, A. Fernández-Villegas, M. Robbins, J. Gurke, E. J. Moonen, G. S. Kaminski Schierle, G. G. Malliaras, Advanced Science **2021**, 8, 13 2004434.

Caption of Supplementary Video

Video S1: Video of concurrent calcium imaging and electrophysiology recording corresponding to data presented in Figure 6b (5x speed).
